# Intracellular Ca^2+^ release decelerates mitochondrial cristae dynamics within the junctions to the endoplasmic reticulum

**DOI:** 10.1007/s00424-018-2133-0

**Published:** 2018-03-12

**Authors:** Benjamin Gottschalk, Christinae Klec, Markus Waldeck-Weiermair, Roland Malli, Wolfgang F. Graier

**Affiliations:** 1 Molecular Biology and Biochemistry, Gottfried Schatz Research Center, Medical University of Graz, Neue Stiftingtalstraße 6/6, 8010 Graz, Austria; 2 BioTechMed, Graz, Austria

**Keywords:** Cristae dynamics, Microscopy, Mitochondria, Mitochondrial Ca^2+^, Mitochondria-associated membranes, OPA1, MCU, Structured illumination microscopy

## Abstract

Mitochondria are multifunctional organelles that essentially contribute to cell signaling by sophisticated mechanisms of communications. Live cell imaging studies showed that mitochondria are dynamic and complex structures that form ramified networks by directed movements, fission, and fusion events. There is emerging evidence that the morphology of mitochondria determines cellular functions and vice versa. Several intracellular signaling pathways and messengers including Ca^2+^ dynamically influence the architecture of mitochondria. Because electron microscopy cannot be utilized for an assessment of dynamics of mitochondrial morphology in intact cells, most studies were performed using wide-field or laser confocal fluorescence microscopies that, due to limitations of their spatial resolution, do not allow investigating sub-mitochondrial structures. Accordingly, our understanding of the dynamics of substructures of mitochondria is quite limited. Here, we present a robust super-resolution method to quantify the dynamics of mitochondrial cristae, the main substructures of the inner mitochondrial membrane, exploiting structured illumination microscopy (SIM). We observed that knockdown of the dynamin-like 120-kDa protein, which is encoded by the OPA1 gene, specifically reduces the dynamics of the mitochondrial cristae membranes (CM), while the inner boundary membrane (IBM) remained flexible. We further used dual color SIM to quantify the dynamics of CM in the junction between mitochondria and the endoplasmic reticulum (ER; mitochondrial associated membranes, MAMs). Intracellular Ca^2+^ release spatially reduced CM-dynamics in MAMs. Moreover, CM-dynamics was independent from matrix Ca^2+^ signal. Our data suggest that local Ca^2+^ signals specifically control CM-dynamics and structure to facilitate a well-balanced functional (Ca^2+^) interplay between mitochondria and the ER.

**Electronic supplementary material:**

The online version of this article (10.1007/s00424-018-2133-0) contains supplementary material, which is available to authorized users.

## Introduction

The network of mitochondria in living cells represents a highly dynamic structure undergoing continuous fission and fusion events. In addition, most mitochondria move within cells mainly via motor proteins attached to the cytoskeleton [4, 23, 36]. Likewise, the mitochondrial ultrastructure and sub-mitochondrial morphology is steadily changing [19, 21, 37]. While the outer mitochondrial membrane (OMM) forms a rather uniform, unstructured shell, the surface of the inner mitochondrial membrane (IMM) is further organized and separated in the cristae membrane (CM), representing the many invaginations of the IMM into the mitochondrial matrix, and the inner boundary membrane (IBM) directly facing the OMM. The CM and IBM compartments are separated via small, tubular connections called cristae junctions (CJ) [17, 26]. Dynamic remodeling of the IMM with its various compartments is known to be involved into apoptosis, which is induced by the release of cytochrome C from the cristae upon morphological changes of the CM and CJ [39]. In addition, the ATP synthase complex plays an essential role in cristae modeling. Dimerization of the ATP synthase and induction of membrane curvature is mandatory for normal cristae morphology [32]. The dynamin-related GTPase OPA1 (Optical Atrophy 1) is located to the IMM and controls the diameter of CJ, thus being crucially involved in cytochrome c release and, hence, the initiation of apoptosis [39]. Notably, it was shown that the oligomerization of membrane-bound and the soluble forms of OPA1 in the inner mitochondrial space (IMS) correlates with the tightness of CJ [11, 14, 35]. Accordingly, a depletion of OPA1 leads to fragmentation of mitochondria and also evokes drastic disorganization of the cristae [1, 5, 22] and increased susceptibility to apoptosis [24]. Moreover, cristae shape, respiratory chain super-complexes, complex-I-dependent respiration, and respiratory growth are impaired after OPA1 depletion [6]. OPA1 might also play a role in mitochondrial Ca^2+^ signaling as knock down of OPA1 enhances Ca^2+^ influx into mitochondria by increasing the access of Ca^2+^ to the transporters in the cristae membrane [12].

Mitochondria are often in close proximity to the endoplasmic reticulum (ER) and interorganellar tethers are established by various proteins like mitofusin 2, the inositol 1,4,5-trisphosphate receptors (IP_3_R-Grp75-VDAC complex [33]), or VDACs (voltage-dependent anion channels) [8, 30]. This close proximity of both organelles enables efficient Ca^2+^ transfer upon IP_3_-mediated ER Ca^2+^ release to mitochondria [16]. Contact sides of mitochondria and the ER, the so-called mitochondria-associated membranes (MAMs), play an important role in signal transduction and Ca^2+^ signaling [9, 16].

Until recently, an accurate visualization and quantification of these important organelle contact sides and the ultrastructure of mitochondria was only feasible in fixed cells using electron microscopy approaches. However, recently, various super-resolution fluorescence imaging approaches substantially surpass the diffraction limit and allow a far better visualization of organelles in living cells. In this study, we utilized structured illumination microscopy (SIM) that uses a shifting and rotating grid pattern to illuminate the sample plane at various optical angles. This allows the analysis and subtraction of the moiré pattern that surpasses the Abbe diffraction limit by about twofold [15, 29]. Recently, several groups were able to resolve the inner mitochondrial structure with 3D-SIM but a quantification approach was not published so far [10, 31, 38]. Because the size of the IMM structures is below the Abbe diffraction limit and cannot be resolved with conventional microscopy, a customized N-SIM System was used in this study to visualize the structure/function relations involved in the dynamics of subdomains of the IMM.

## Material and methods

### SIM imaging

The N-SIM-setup (Nikon Austria, Vienna) used is equipped with 405-, 488-, 515-, 532-, and 561-nm excitation lasers introduced at the back focal plane inside the SIM box with a multimodal optical fiber. For super-resolution, a CFI SR Apochromat TIRF 100×-oil (NA 1.49) objective was mounted on a Nikon-Structured Illumination Microscopy (N-SIM®) System with standard wide field and SIM filter sets and equipped with an Andor iXon3® EMCCD camera. For calibration and reconstruction of SIM images, the Nikon software Nis-Elements was used. Reconstruction was always done with the same robust setting to avoid artifact generation and ensures reproducibility with a small loss of resolution of 10% compared to most sensitive and rigorous reconstruction settings. The general obtained resolution (full width at half maximum; FWHM) was approximately 120 to 130 nm measured with fluorescent 100-nm Tetraspec beads (Invitrogen™, Thermo Fisher Scientific, Vienna, Austria).

Prior to each measurement, laser adjustment was checked by projecting the laser beam through the objective at the top cover of the bright field arm of the microscope. Lasers were aligned and focused using the interlock system screws at the N-SIM box to ensure appropriate illumination of the sample. The 100-nm Tetraspec beads (Invitrogen™, Thermo Fisher Scientific, Vienna, Austria) were diluted 1:200 in 1 ml 0.01% poly-l-lysine and incubated on the 1.5H high-precision glass coverslips (Marienfeld-Superior, Lauda Königshofen, Germany) for 20 min. Afterwards, the plate was washed once and transferred to the live cell camber containing 1 ml of buffer (2CaB) containing the following (in mM): 2 CaCl_2_, 140 NaCl, 5 KCl, 1 MgCl_2_, 1 HEPES, and 10 D-Glucose; pH was adjusted to 7.4 (all buffer salts were obtained from Roth, Graz, Austria). Ring correction was done as follows: 3D-stacks of beads were acquired to verify the point spread function of the system. Potential asymmetric point spread function was corrected using the objective correction ring. Subsequently, grating block adjustment was performed to find the perfect focus for the laser beam interference at the focal plane. This process was automatically carried out by the Nikon software.

### Cell culture

HeLa cells (ATCC® CCL-2.2™) were seeded on 1.5H high-precision glass coverslips (Marienfeld-Superior) and cultured in DMEM (D5523, Sigma-Aldrich, Darmstadt, Germany) containing 10% FCS (Gibco™, Thermo Fisher Scientific), penicillin (100 U/ml), streptomycin (100 μg/ml), and amphotericin B (1.25 μg/ml) (all Gibco™) in a humidified incubator (37 °C, 5% CO_2_/95% air).

### Mitochondria staining

Cells were washed once with loading-buffer containing the following (in mM): 2 CaCl2, 135 NaCl, 5 KCl, 1 MgCl_2_, 1 HEPES, 2.6 NaHCO_3_, 0.44 KH_2_PO_4_, 0.34 Na_2_HPO_4_, 10 D-glucose (Roth), 0.1% vitamins, 0.2% essential amino acids, and 1% penicillin/streptomycin (Gibco™) at pH 7.4). Cells were incubated in loading-buffer containing 0.5 μM MTG for 40 min. Thereafter, cells were washed once with loading buffer and imaged in 2CaB.

### Transfection and siRNA treatment

HeLa cells were grown under standard culture conditions until 50% confluence was reached and transfected in DMEM (without FCS and antibiotics) with 1.5 μg/ml plasmids and/or 100 nM of the respective siRNA with 2.5 μg/ml TransFast™ transfection reagent (Promega, Madison, WI, USA). After 24 h, the medium was replaced with DMEM containing 10% FCS and penicillin and streptomycin and kept for further 24 h prior the experiments. The specific siRNAs (Microsynth) used in this study are listed in Supplementary Table 1.

### Quantification of the IMM-kinetic

Live SIM imaging sequences of 30 frames with a frame rate of 1 Hz of MTG-stained HeLa cells were recorded. The open-source program ImageJ (Fiji) was used to analyze the CM-kinetics. First, time-lapse acquisitions were background corrected using a histogram-based background subtractor (Mosaic group). Further, a time-dependent bleach correction using an exponential fitting method was applied to correct for intensity decrease over time caused by photo bleaching. The corrected time lapse was auto thresholded twice. A global Otsu auto threshold [25] was used to segment mitochondria and a local mean auto threshold with a two-pixel radius was used to identify small intensity differences within the IMM structure. By pixelwise multiplying both thresholded time lapses, the structural information of the IMM can be revealed. To determine the IMM-kinetic the difference of subsequent frames in time was calculated. Since mitochondria are moving very fast and changing their shape, the changes per frame of the IBM shell were excluded. Therefore, a closing algorithm consisting in the succession of a dilation with an erosion (iterations = 2 count = 2) and a whole filling algorithm were applied on the thresholded time lapse containing the IMM-structural information. Subsequent frames were subtracted from each other to get the IBM-kinetic of the mitochondrial shell. By subtracting the difference per frame of the IBM mitochondrial shell from the overall IMM-changes within the mitochondria, the CM-kinetics were extracted. Afterwards, the accumulated pixel counts of the CM-changes were normalized to the global Otzu auto thresholded pixel count for each frame and the mean of the time lapse was determined.

### Quantification of the CM-kinetic in relation to ER proximity

HeLa cells transfected with ER-RFP and stained with MTG were recorded with live dual color SIM imaging. Three repeats of one frame ER-targeted red fluorescence protein (ER-RFP) and 10 frames MTG at a frequency of 1 Hz were recorded. To quantify the inner mitochondrial membrane kinetic in correlation to the sub mitochondrial localization, the IMM-kinetics were measured like described above. Additionally, ER-RFP was globally Otzu thresholded. The overlap of both labels was generated by framewise multiplication and defined as mitochondrial-associated membrane (MAM). These regions were enlarged by stepwise dilatation (iterations = 5, 10, and 20; pixel count = 3) to increase the area. By framewise multiplication of these differently enlarged MAM regions with the CM-inter-frame changes, the IMM-kinetic in close proximity to the ER could be defined. Further, the auto Otzu thresholded MTG area was reduced by multiplication with the differently enlarged MAM regions to get the proper size for normalization.

### mRNA isolation and real-time PCR

Total cellular RNA was isolated from HeLa cells using the PEQLAB total RNA isolation kit (Peqlab, Erlangen, Germany), followed by reverse transcription to cDNA, performed in a thermal cycler (Peqlab) using the high-capacity cDNA reverse transcription kit (Applied Biosystems, Foster City, USA). The qPCR reaction was set up with the GoTag® qPCR Master Mix (Promega, Mannheim, Germany) together with gene-specific primers (Invitrogen, Vienna, Austria). Experiments were performed on a LightCycler 480 (Roche Diagnostics, Vienna, Austria). Relative expression of specific genes was normalized to human GAPDH, as a reference gene. Primer sequences are as follows: Opa1 for: 5’-GGTTGGAGATCAGAGTGCTGG-3′, Opa1 rev: 5’-GGACCTTCACTCAGAGTCACC-3′, MCU for: 5’-TTCCTGGCAGAATTTGGGAG-3′, MCU rev: 5’-AGAGATAGGCTTGAGTGTGAAC-3′, GAPDH (QuantiTect® Primer Assay Hs_GAPDH, Qiagen, Hilden, Germany).

### Western blots

Western blots were performed according to standard protocols. Briefly, cell lysis was conducted with RIPA buffer (Bio-Rad formulation) supplemented with protease inhibitor cocktail (1:50; Sigma Aldrich, Vienna, Austria), followed by sonication (80% amplitude, 2 × 15 sec). Samples were denatured in 1 × Laemmli sample buffer and resolved on a 7.5% or 12.5% SDS-PAGE gel together with PageRuler™ Plus Prestained Protein Ladder (Fisher Scientific, Vienna Austria). Blots were blocked and antibodies diluted in 5% BSA (Sigma Aldrich) in TBS-T. The following antibodies were used: MCU (D2Z3B, 1:1000, Cell Signaling Technology, MA, USA), Opa1 (D6U6N, 1:1000**,** Cell Signaling Technology) and β-actin (sc-47778, 1:500, Santa Cruz Biotechnology, Heidelberg, Germany). HRP labeled anti-mouse (PI-2000, 1:1000, Vector Laboratories, Burlingame, USA) and anti-rabbit (sc-2357, 1:1000, Santa Cruz Biotechnologies) were used as secondary antibodies. For visualization, the SuperSignal West Pico PLUS kit (Fisher Scientific) was used and detection was conducted on the ChemiDoc System (Bio-Rad Laboratories, Vienna, Austria).

### Statistical analysis and reproducibility

Each exact *n* value and the number of independent experiments are indicated in each figure legend. Statistical analysis was performed using the GraphPad Prism software version 5.04 (GraphPad Software, San Diego, CA, USA) or Microsoft Excel. Analysis of variance and *t* test were used for evaluation of the statistical significance. *P* values are indicated within the figures, and *P* < 0.05 was considered to be significant.

### Data availability

All source data for the preparation of this manuscript are available from the authors on request.

## Results

### Super-resolution SIM allows the discrimination of the very kinetics of the IBM vs. CM

**Fig. 1:**
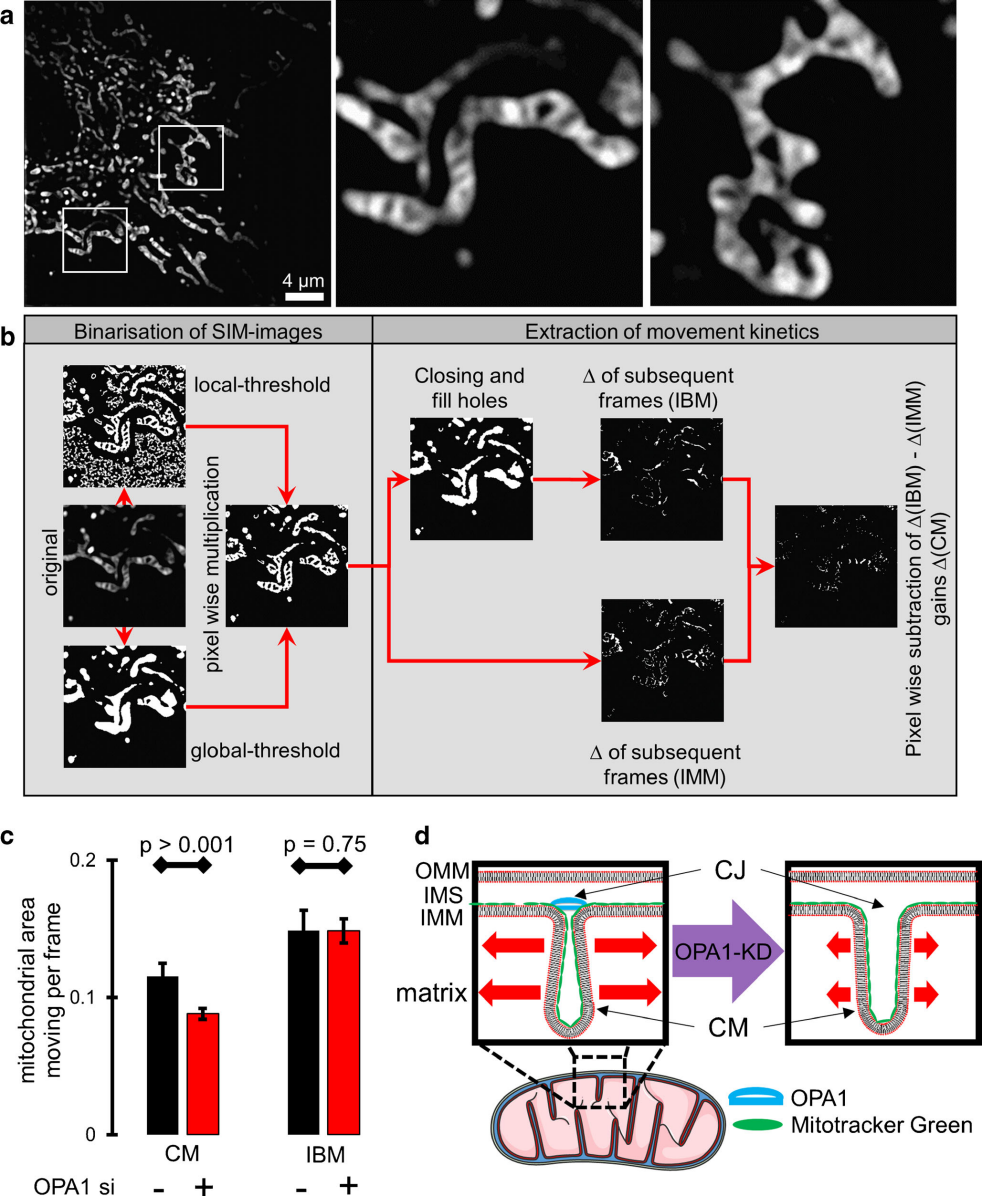
Validating a method to quantify IMM-kinetics applying super-resolution N-SIM. **a** HeLa cells stained for MTG were imaged with live N-SIM at 1 Hz. The corresponding video is available within Supplementary Video 1. **b** Process applied to quantify the kinetics of the IMM. Time-lapse images were binarized applying locally auto thresholding (*left panel*), a closing and fill holes algorithm was applied and the delta intensity of subsequent frames were detected and determined as mitochondrial IBM-changes (*middle panel*). Delta intensities of subsequent frames without applying the closing and fill holes algorithm determined the mitochondrial IMM-changes. IBM-changes were subtracted for IMM-changes revealing the dynamics of the CM. Finally, the overall pixel intensities of CM-changes were normalized to the global threshold area presenting the percentage of moving area inside the mitochondria. **c** The percentage of mitochondrial area moving per frame separated in the CM and IBM was quantified for control siRNA and OPA1 siRNA (*n* = 6). **d** Schematic representation of the IMM and the OPA1-controlled CJ. Diminution of OPA1 leads to widened CJ and reduced cristae kinetics. Images and analyses were obtained from at least five cells in each of six experiments. Bars represent mean ± SEM


Initially, we verified the feasibility of our attempt to determine and quantify the dynamics of the IMM by utilizing SIM. Therefore, HeLa cells were loaded with MitoTracker Green® (MTG) and directly imaged at a frequency of 1 Hz with SIM. Live SIM time-lapse imaging allowed a detailed analysis of the dynamic changes of mitochondrial membranes (Fig. 1a, Suppl. Video 1).

(MP4 3019 kb)


To determine the cristae dynamics under various conditions, we developed an analysis strategy to filter and quantify the dynamic movement of the CM separately to that of the IBM. This strategy consists of the following five steps (Fig. 1b): *First*, images out of a time-lapse acquisition were binarized applying locally auto thresholding. *Second*, to extract the individual kinetics of the IMM and CM, a closing and fill holes algorithm was applied and the delta intensity of subsequent frames were detected and determined as mitochondrial IBM-changes. *Third*, the delta intensities of subsequent frames without applying the closing and fill holes algorithm were detected and determined as mitochondrial IMM-changes. *Fourth*, IBM-changes were subtracted for IMM-changes revealing the changes within the CM (i.e., CM-changes). *Fifth*, the overall pixel intensities of CM-changes were framewise normalized to the global threshold area to get the percentage of moving mitochondrial area inside the mitochondria.

### Depletion of OPA1 specifically affects cristae dynamics but not that of the IBM

To test the sensitivity of the SIM-based analysis of spatial IMM-dynamics described above, HeLa cells were depleted from OPA1 a known protein of the CJ by transient transfection with the respective siRNA (for knock-down efficiency please see Suppl. Figure 1). Our analysis of spatial IMM kinetics revealed that the knock-down of OPA1 exclusively decreased kinetics of the CM while that of the IBM remained unchanged **(**Fig. 1c). Considering that a knock-down of OPA1 results in a broadening of the CJ [11, 14], our data suggest that cristae dynamics is reduced under conditions of widened CJ (Fig. 1d).

### Analyzing the spatial IMM-kinetic in proximity to MAM structures

**Fig. 2:**
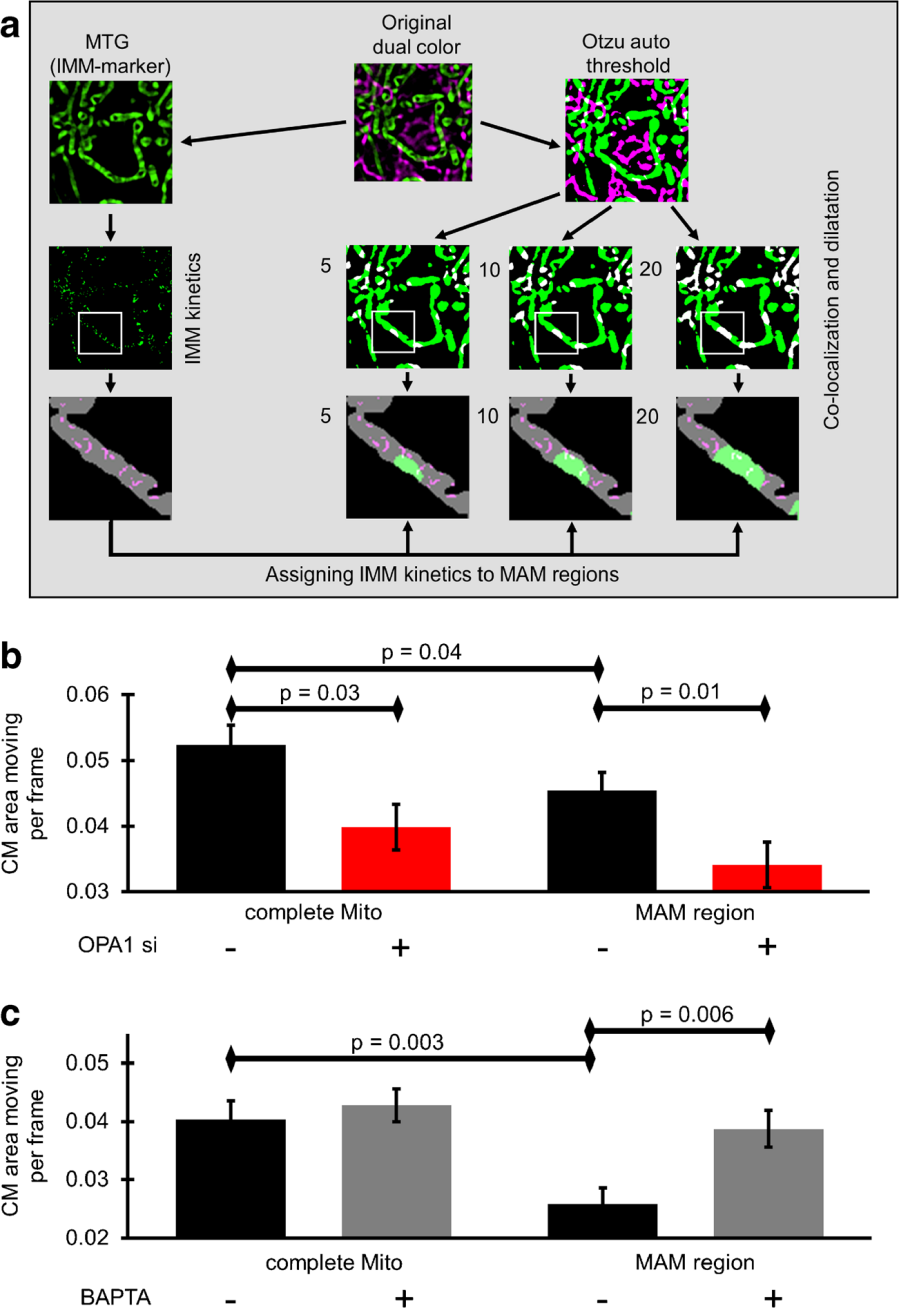
Localizing ER-mitochondrial-associated membranes (MAMs) and verification of the IMM-dynamics in correlation to the proximity/distance to MAMs. **a** HeLa cells were transfected with ER-RFP, stained with MTG and imaged with live N-SIM. Both channels were auto Otzu thresholded. Overlap regions of ER and mitochondria were determined as MAMs and the areas of co-localization were incrementally dilated using 5, 10, or 20 iterations. The IMM-dynamics measured according to Fig. 1b were cropped to the dilated areas and subsequently quantified. **b** HeLa cells transfected with ER-RFP and control siRNA or OPA1-specific siRNA, stained with MTG, were imaged with live N-SIM. CM-dynamics over the entire mitochondrion and in MAM-close regions (five iterations) were quantified. Images and analyses were obtained from at least five cells in each of eight experiments. **c** HeLa cells stained with MTG and transfected with ER-RFP were imaged with N-SIM. Cells were incubated for 45 min in 2Ca-buffer or 0Ca-buffer with 50 μM BAPTA-AM. CM-dynamics of the complete mitochondria and that of MAM-related areas (five iterations) were quantified. Images and analyses were obtained from at least five cells in each of six experiments. Bars represent mean ± SEM

To investigate whether or not sites of physical and function ER-mitochondrial coupling (i.e., MAMs) influence the spatial IMM kinetics in their neighborhood, HeLa cells were transfected with ER-targeted red fluorescence protein (ER-RFP) and stained with MTG. The co-localization of both auto thresholded labels obtained in the SIM was defined as MAMs. Spatial IMM-kinetics were measured within distinct areas with a given proximity to MAM structures experimentally defined as incremental dilatations of the defined MAM area (Fig. 2a). Expressing ER-RFP and labeling mitochondria with MTG (Suppl. Figure 2) allowed us to localize MAMs and to quantify IMM-dynamics to draw a correlation between CM-dynamics and its distance/proximity to MAMs. Using this approach, we could show significantly lower CM-kinetics in close proximity to MAMs compared to the overall CM-dynamics of the entire mitochondrial IMM area (Fig. 2b). Furthermore, OPA1 knock-down resulted in a significant reduction of the CM-dynamics independently from its proximity to existing MAMs (Fig. 2b). To test the involvement of Ca^2+^ hotspots within the MAMs to locally regulate CM-dynamics, spatial Ca^2+^ signaling was prevented by loading cells with the fast Ca^2+^ chelator BAPTA/AM and CM-dynamics was measured. Chelation of MAM-Ca^2+^ prevented the reduction of CM-dynamics in the proximity of MAMs and restored its dynamics to levels of the complete mitochondria (Fig. 2c).

### ER Ca^2+^ release spatially influences CM-kinetics

**Fig. 3:**
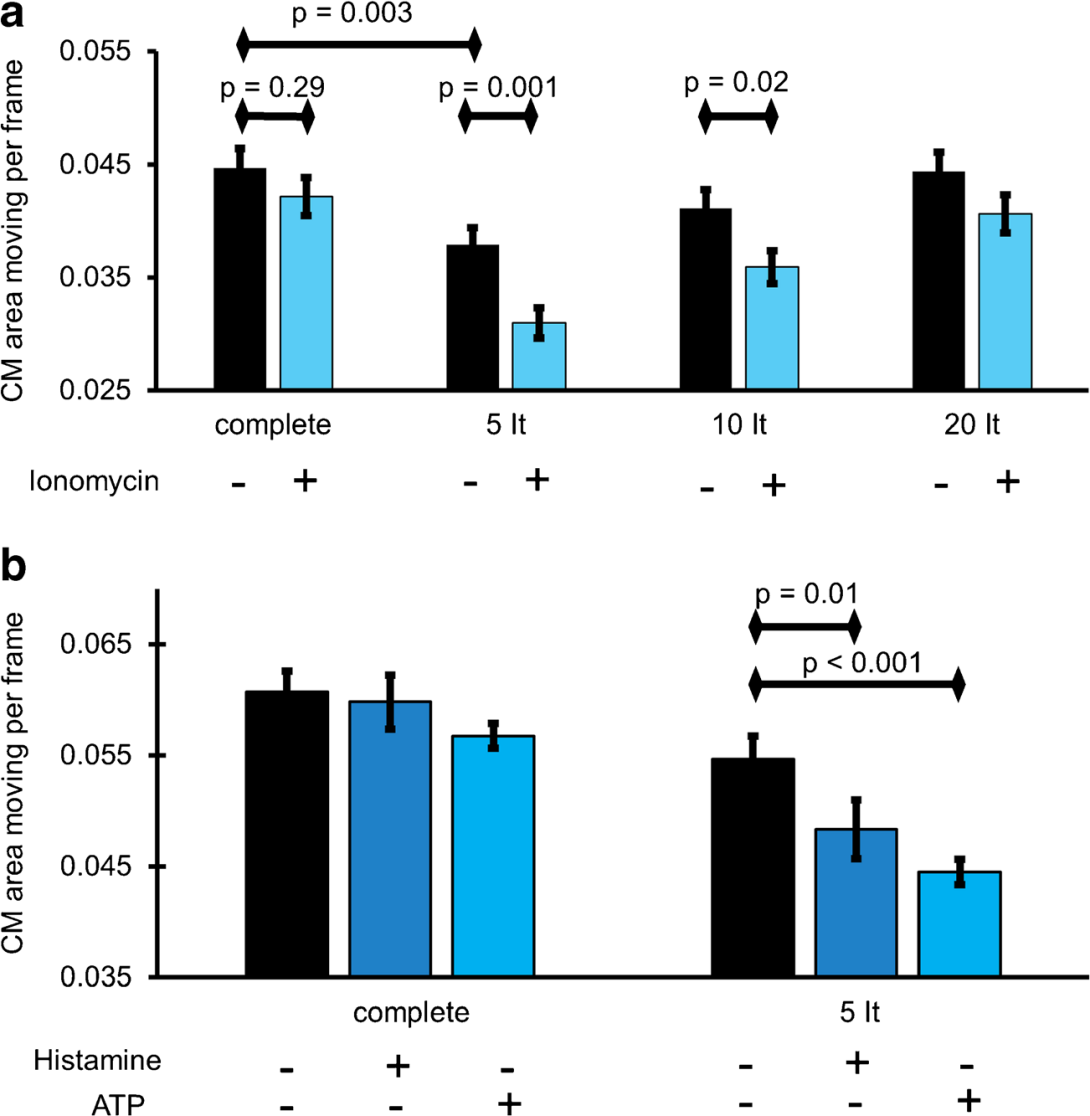
Differential IMM-dynamics in MAM and non-MAM mitochondrial regions upon **a** ionomycin- or **b** agonist-induced ER Ca^2+^ release. **a** HeLa cells transfected with ER-RFP and stained with MTG were imaged with live N-SIM. Cells were treated with 200 nM ionomycin to induce selective ER Ca^2+^ release. IMM-dynamics was measured within the whole IMM surface and compared with that in close proximity to the MAMs with increasing area of influence, represented by the increasing amount of dilatation of 5, 10, and 20 iterations. Images and analyses were obtained from at least five cells in each of eight experiments. **b** HeLa cells transfected with ER-RFP and stained with MTG were treated with histamine (100 μM) or ATP (100 μM) to induce ER Ca^2+^ release. CM-dynamics of the whole IMM surface as well as the area in close proximity to MAMs (five iterations) were quantified. Images and analyses were obtained from at least five cells in each of eight experiments. Bars represent mean ± SEM

To test the effect of intracellular Ca^2+^ release on CM-dynamics, cells were transfected with ER-RFP and stained with MTG. Ionomycin (200 nM) was used to evoke global Ca^2+^ release from the ER [2, 3]. Cells were imaged with and without the addition of ionomycin. While the CM-dynamics within the whole mitochondrion remained unchanged after the global Ca^2+^ release, the kinetics of CM decreased with the proximity (i.e., 5 and 10 iterations) to MAMs (Fig. 3a). In further distance from the MAM structures (i.e., 20 iterations), kinetics of CM was comparable to that of the whole mitochondria (Fig. 3a).

Next, we investigated the impact of agonist-induced ER Ca^2+^ release on CM-dynamics in the entire mitochondria and in correlation with the distance to MAMs. Therefore, HeLa cells that were transfected with ER-RFP and stained with MTG were stimulated with the IP_3_-generating agonists histamine or ATP to induce strong ER-Ca^2+^ release. Similar to our data with ionomycin, the CM-dynamics of the entire mitochondria was not affected upon strong IP_3_-mediated ER Ca^2−^ release (Fig. 3b). However, CM-dynamics in the proximity of MAMs was strongly attenuated upon agonist-induced intracellular Ca^2+^ release, independently of the agonist used (Fig. 3b).

### No further Ca^2+^-induced CM deceleration was found under OPA1 knock-down

**Fig. 4:**
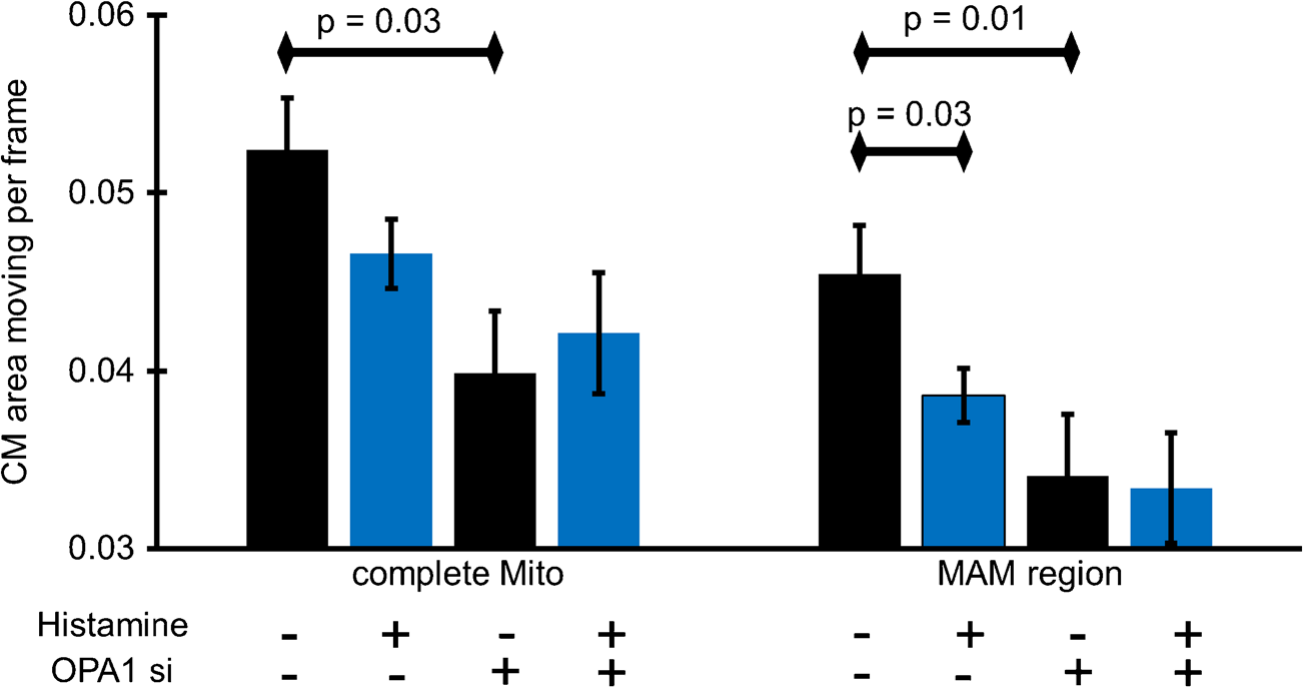
Impact of agonist-induced ER Ca^2+^ release on IMM-dynamics in control cells and cells depleted from OPA1. HeLa cells stained with MTG and transfected with ER-RFP and either control siRNA or OPA1-specific siRNA were imaged with N-SIM. Histamine (100 μM) was added to induce ER Ca^2+^ release. CM-dynamics of the entire IMM surface and that of MAM-related areas (five iterations) were quantified. Images and analyses were obtained from at least five cells in each of eight experiments. Bars represent mean ± SEM

Furthermore, we investigated the influence of intracellular Ca^2+^ release on the CM-dynamics in cells depleted from OPA1. The knock-down of OPA1 in HeLa cells reduced the general dynamics in MAM-close and MAM-distinct CM (see also Fig. 2b and 4), while no further effect of agonist-induced intracellular Ca^2+^ release on CM-dynamics was observed on the entire mitochondrion (Fig. 4). Hence, in OPA1-depleted cells, histamine-induced ER Ca^2+^ release did not affect mitochondrial branching (i.e., form factor; Suppl. Figure 3). However, mitochondrial shape changed upon histamine stimulation independently of OPA1 expression (i.e., aspect ratio; Suppl. Figure 3). These data suggest that a general deceleration of the CM-dynamics in the entire mitochondrion (which is due to the broadening of the CJ induced by the lack of OPA1) cannot be further affected by intracellularly released Ca^2+^.

### Ca^2+^-induced deceleration of MAM-close CM did not require mitochondrial Ca^2+^ uptake

**Fig. 5:**
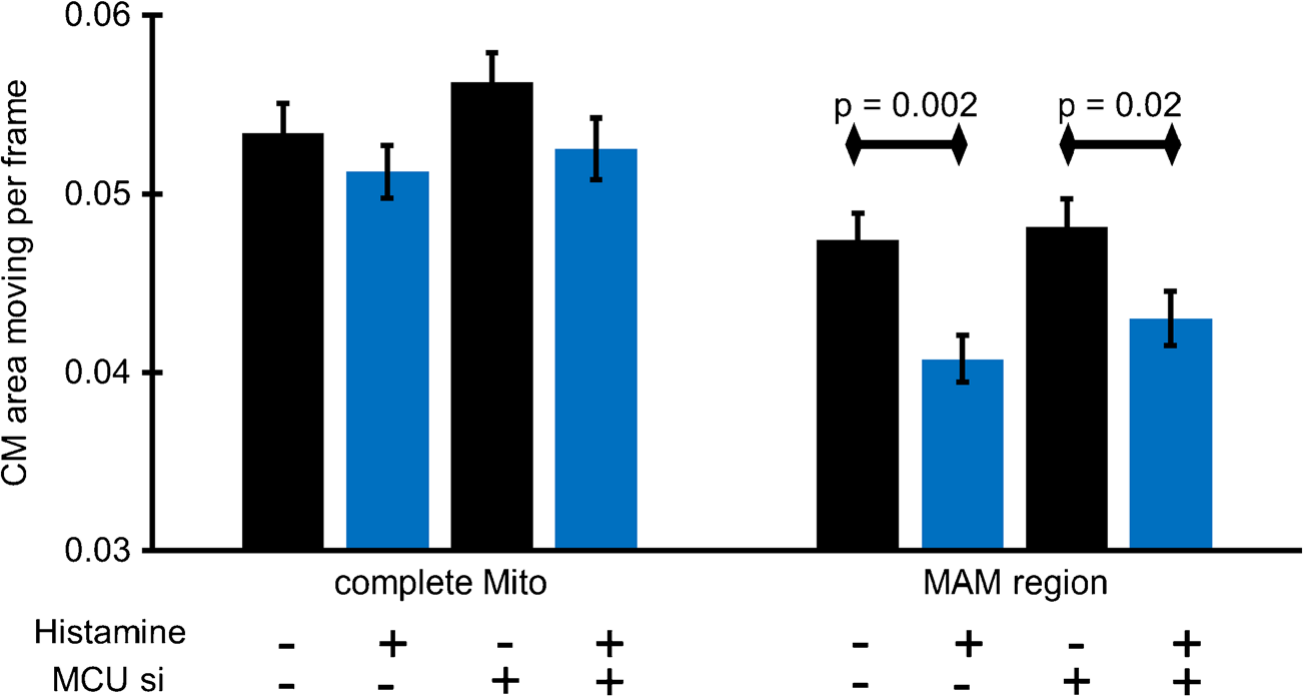
Elevations in matrix Ca^2+^ are not involved in ER-Ca^2+^-release-mediated deceleration of CM in the proximity of MAMs. HeLa cells stained with MTG and transfected with either ER-RFP and control siRNA or MCU-specific siRNA were imaged with N-SIM. Histamine (100 μM) was added to induce ER Ca^2+^ release. CM-kinetics of the entire IMM surface and that of MAM-related area (five iterations) were quantified. Images and analyses were obtained from at least five cells in each of eight experiments. Bars represent mean ± SEM

To verify whether or not mitochondrial Ca^2+^ uptake is required for the decrease in the kinetics of MAM-close CM, mitochondrial Ca^2+^ influx was prevented by siRNA-mediated diminution of the expression of MCU [20] (for knock-down efficiency, please see Suppl. Figure 1). Knock-down of MCU had no impact on the effect of intracellularly released Ca^2+^ on global mitochondrial and MAM-related CM-dynamics (Fig. 5). These findings indicate that the Ca^2+^-induced inhibition of MAM-close CM-kinetics is independent of mitochondrial Ca^2+^ uptake. Hence, depletion of MCU did not affect mitochondrial shape (i.e., aspect ratio) and histamine influenced mitochondrial branching (i.e., form factor; Suppl. Figure 4), thus, indicating that Ca^2+^-induced changes in mitochondrial shape do not require Ca^2+^ influx in the organelle.

## Discussion

In this study, we used an optimized SIM system and a newly designed super-resolution image analysis approach to visualize and quantify the dynamics of the main subdomains of the mitochondria, the CM and IBM, of the IMM in intact living cells. Our data demonstrate that both the proximity of mitochondria to the ER and Ca^2+^ release significantly and specifically affect the dynamics of the CM, while matrix Ca^2+^ does not impact mitochondrial membrane dynamics.

Mitochondria are very dynamic and interactive organelles that feature two very specialized membranes, the OMM, representing the shelter against the cytosol, and the IMM that consists of the IBM and the CM divided by the CJ. Very recently, the sub-structure of mitochondria gained much attention due to reports that indicated that specific functions of IMM-localized proteins and protein complexes are facilitated by their distinct distribution within various segments of the IMM. In particular, complexes of the respiratory chain have been described to localize almost exclusively in the CM while other (presumably) ion channels and transporters, like UCP4, were found across the CJ in the IBM [18]. As the distinct distribution of proteins and their complexes within the various segments of the IMM strongly correlates with their biological functions, the position in respect to the most interacting neighbor of the mitochondria, the ER, is of utmost importance. However, in view of the strong dynamics of mitochondria, one might expect that ER-mitochondria junctions master the dynamics of IMM yielding a perfect match in the orientation of ER and mitochondrial exchange machineries. Accordingly, the exact positioning of the every segment of the IMM and their ultrastructure is like to be important for the organelle’s function, fate, and subsequently, cell survival. Therefore, we intended to visualize, measure, and subsequently analyze the dynamics of the IMM segments in correlation to ER contact sides applying the sophisticated SIM technology [10, 38]. Moreover, we were able to study the impact of Ca^2+^ on the dynamics of the CM and IBM using this super-resolution imaging approach. The combination of optimized SIM microscopy with a organelle-specific fluorophore, organelle-targeted fluorescent protein, and our imaging processing strategy allowed us to resolve dynamics of organellar sub-compartments on the super-resolution level in intact living cells.

The critical importance of an optimized mitochondrial ultrastructure for the organelle’s fate is exemplarily illustrated by OPA1, which determines the width of the CM due to its oligomerization of membrane-bound and soluble forms [11, 14, 35]. Notably, a loss of OPA1 leads to opening of the CJ allowing Ca^2+^ influx from the intermembrane space into the inner cristae. Such Ca^2+^ accumulation inside the cristae is known to accompany altered cristae shape [12, 14], impair respiratory chain super-complexes and respiration [6], and increase the susceptibility of the organelle for apoptotic stimuli [11]. Our present data using a targeted genetically encoded fluorescent protein together with MTG revealed that the dynamics of the CM but not the IBM was strongly reduced by depletion of OPA1. These findings indicate that the widened cristae structure establishes a higher diffusion resistance of the cristae infoldings, thus, yielding reduced CM-dynamics (Fig. 1d). Hence, our findings that knock-down of OPA1 reduced CM-dynamics equally in the entire mitochondrion independently of the proximity/distance to MAMs, point to a global mitochondrial mechanism of OPA1 that is rather independent from contacts to other organelles. Accordingly, we speculate that OPA1 controls CM-dynamics in a Ca^2+^-independent manner by stabilizing CJ [14].

Furthermore, we were able to measure the CM-dynamics in relation to the proximity to MAMs. Our findings that CM-dynamics was reduced in the proximity of MAMs and chelation of Ca^2+^ hotspots by BAPTA normalized CM-dynamics in proximity of MAMs to that of the global mitochondria indicates an involvement of spatially localized Ca^2+^ in the regulation of CM-dynamics within the MAMs even under resting conditions. Hence, we investigated the impact of global and local ER Ca^2+^ release as potential regulator of CM-dynamics. Our findings unveil that ER membrane permeabilization for Ca^2+^ with ionomycin [2, 3] reduce CM-dynamics particularly in the vicinity of MAMs. In line with these findings, ER Ca^2+^ mobilization with either histamine or ATP, two physiological IP_3_-generating agonists, yielded a deceleration of CM. This effect strictly correlated with the proximity to MAM structures. Accordingly, we speculate that Ca^2+^ hotspots in MAMs [13] spatially decelerate CM-dynamics while global cytosolic Ca^2+^ elevations do not affect CM-kinetics. At this stage, we do not know whether the Ca^2+^ hotspots, similar to OPA1 knock-down, widen the cristae structure for more efficient mitochondrial Ca^2+^ uptake and thereby reducing the local IMM-dynamics. However, it is also possible that the phenomenon of spatial CM deceleration is mediated by the physical coupling between the ER and the OMM. This junction might be further strengthened by a Ca^2+^-dependent mechanism [28] and, subsequently, hamper CM-dynamics. Notably, Ca^2+^-dependent mechanism opening the cristae structure within the MAMs and thereby reducing the local IMM-dynamics might be important for establishing efficient mitochondrial Ca^2+^ uptake. Hence, Ca^2+^-triggered widening of the CJ within the MAMs might also contribute to the increased fragmentation of mitochondria by facilitating fission events in addition to other already described mechanisms [7, 34].

Our hypothesis that Ca^2+^ hotspots within the MAMs decelerate CM-dynamics, at least in part, by widening the CJ is further supported by our findings that in cells depleted from OPA1, which per se yields CJ opening [14], no further Ca^2+^-induced deceleration of the dynamics of MAM-close CMs were found. These findings also point to a possible, so far underestimated, importance of a dynamic regulation of the width of CJ that facilitates (ion) signaling within the MAMs upon ER Ca^2+^ release. The importance of intra-junctional Ca^2+^ as spatial regulator for CM-dynamics, especially in the MAMs, is further supported by our findings that the deceleration of the dynamics of MAM-associated CMs remains unaffected in cells that were depleted from MCU. These data suggest that elevations in mitochondrial matrix Ca^2+^ is not involved in the vibrant regulation of CM-dynamics and further supports the role of MAM Ca^2+^ elevation in facilitating inter-organelle (Ca^2+^) communications.

Overall, our present data point to a spatial deceleration of the dynamics of CM specifically within the MAMs upon IP_3_-mediated intracellular Ca^2+^ release. Furthermore, a spatial regulation of the CJ by a Ca^2+^-sensitive and OPA1-independent mechanisms as a so far unknown part of the mitochondrial cristae organization system (MICOS) [27] can be predicted. Thus, this study adds a new perspective to the understanding of the ER to mitochondria communication.

## Electronic supplementary material

ESM 1 (PDF 456 kb)
