# Supplementary material for: Intracellular Ca2+ release decelerates mitochondrial cristae dynamics within the junctions to the endoplasmic reticulum
Source: Pflugers Arch. 2018 Mar 12;470(8):1193–203. doi: 10.1007/s00424-018-2133-0 (PMC6047742; doi:10.1007/s00424-018-2133-0)
Supplement: Supplementary file 2 — (PDF 456 kb) [file 424_2018_2133_MOESM1_ESM.pdf]

Intracellular Ca^{2+} release decelerates mitochondrial cristae dynamics within the junctions to the endoplasmic reticulum

Short title: *Quantification of sub-mitochondrial membrane dynamics using SIM*

Benjamin Gottschalk[1], Christinae Klec[1], Markus Waldeck-Weiermair[1], Roland Malli[1,2] and Wolfgang F. Graier[1,2,*]

[1]Molecular Biology and Biochemistry, Gottfried Schatz Research Center, Medical University of Graz, Neue Stiftingtalstraße 6/6, 8010 Graz, Austria

[2]BioTechMed, Graz, Austria

**Correspondence and requests for material should be addressed to:*

Wolfgang F. Graier, Ph.D.

Tel: +43 316 385 71963

eMail: wolfgang.graier@medunigraz.at

Supplemental Video

Suppl. Video: HeLa cells were stained with MTG for 40 min, washed 1x with Loading Buffer and imaged directly at a frequency of 1Hz with live N-SIM.

Supplementary Figures

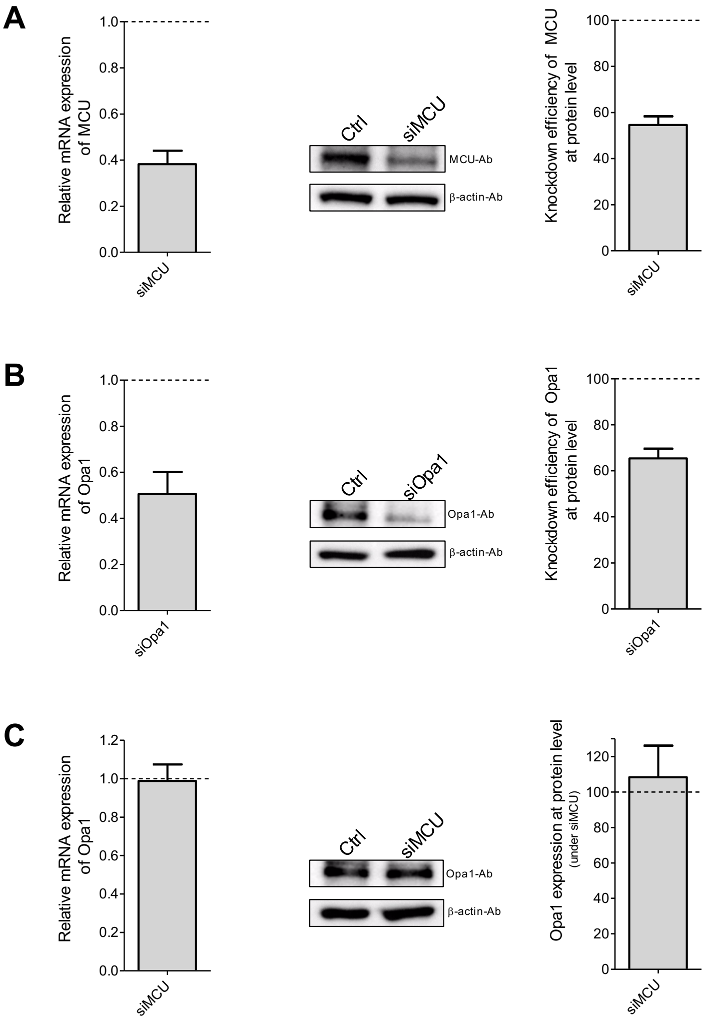

Supplementary Figure 1. Quantification of knockdown efficiency and examination of the influence of MCU knockdown on Opa1 expression level. **(A,B)** Verification of knockdown efficiency of **(A)** MCU and **(B)** Opa1 after treatment with scrambled control siRNA or siRNAs against the specific genes. **(C)** Opa1 expression level analysis under conditions where MCU was knocked out by specific siRNAs. (*Left panels*) quantification of mRNA expression levels by using qRT-PCR, (*middle panels*) representative Western blots, (*right panels*) corresponding statistical analysis of Western blots. For qRT-PCR and Western blot the housekeeping genes GAPDH or β-actin were used, respectively. Bars represent mean ± SEM, n=3.

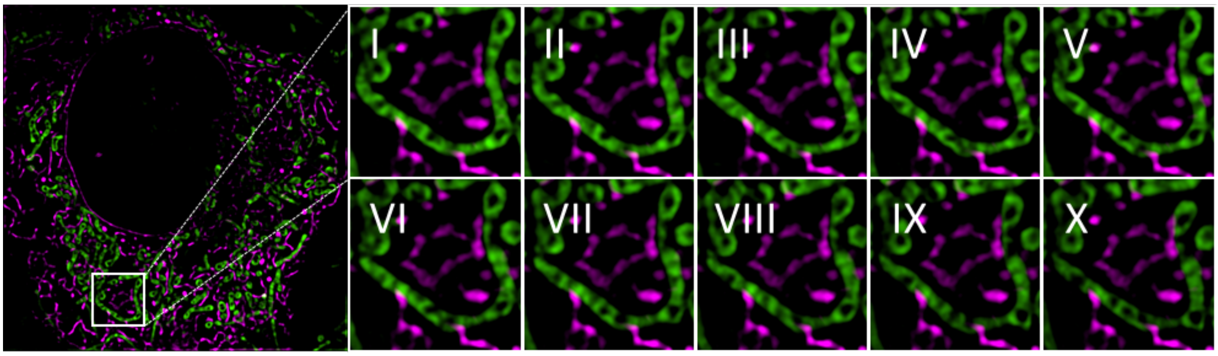

Supplementary Figure 2. HeLa cells transfected with ER-RFP (magenta) and stained 40 min with MTG (green) were imaged. A representative sequence of 10 frames (1 Hz) is shown as a magnification.

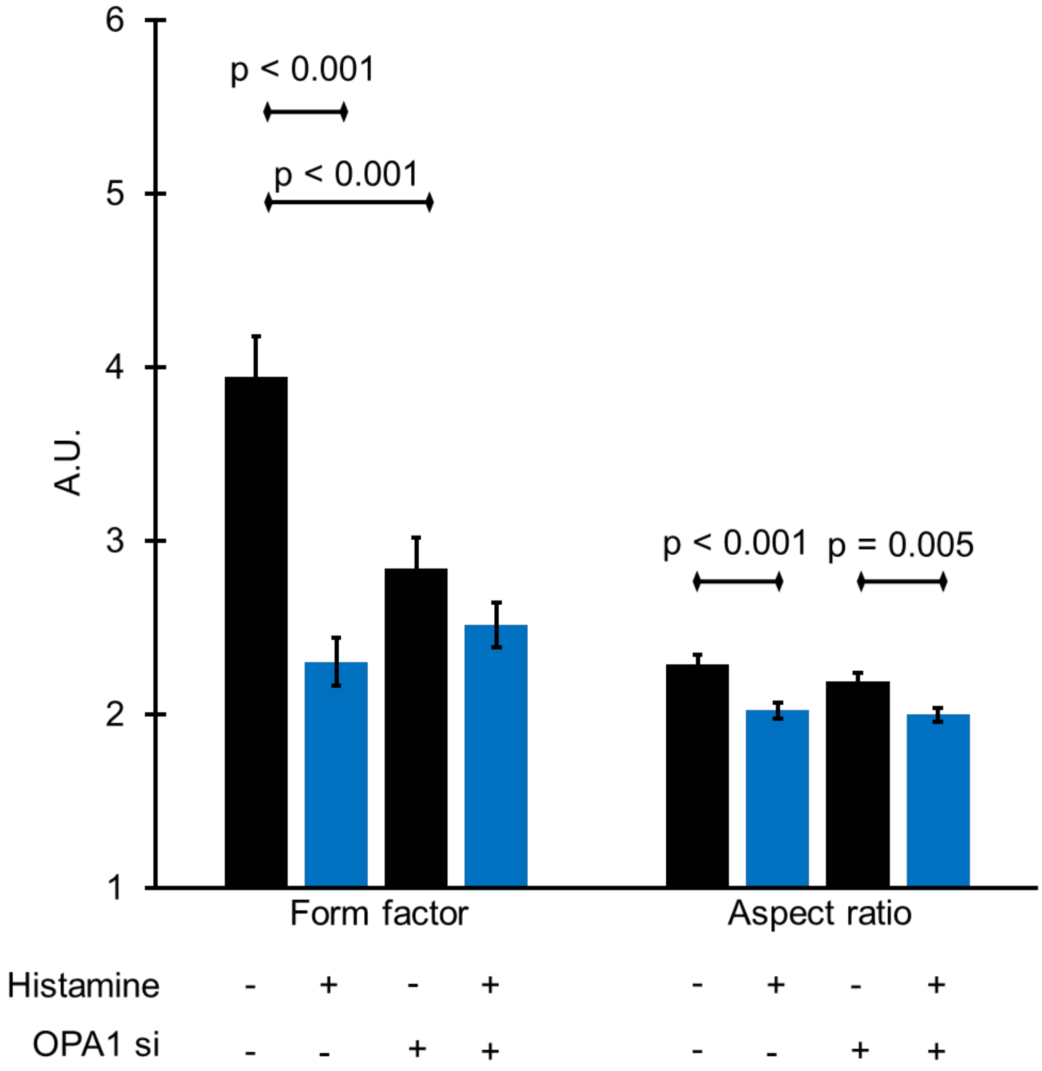

Supplementary Figure 3. HeLa cells stained with MTG and transfected with ER-RFP and control or OPA1-specific siRNAs were imaged with N-SIM. Histamine (100 μM) was added to induce ER Ca^{2+} release. Form factor and aspect ratio were determined using ImageJ. Images and analyses were obtained from at least 5 cells in each of 8 experiments. Bars represent mean ± SEM.

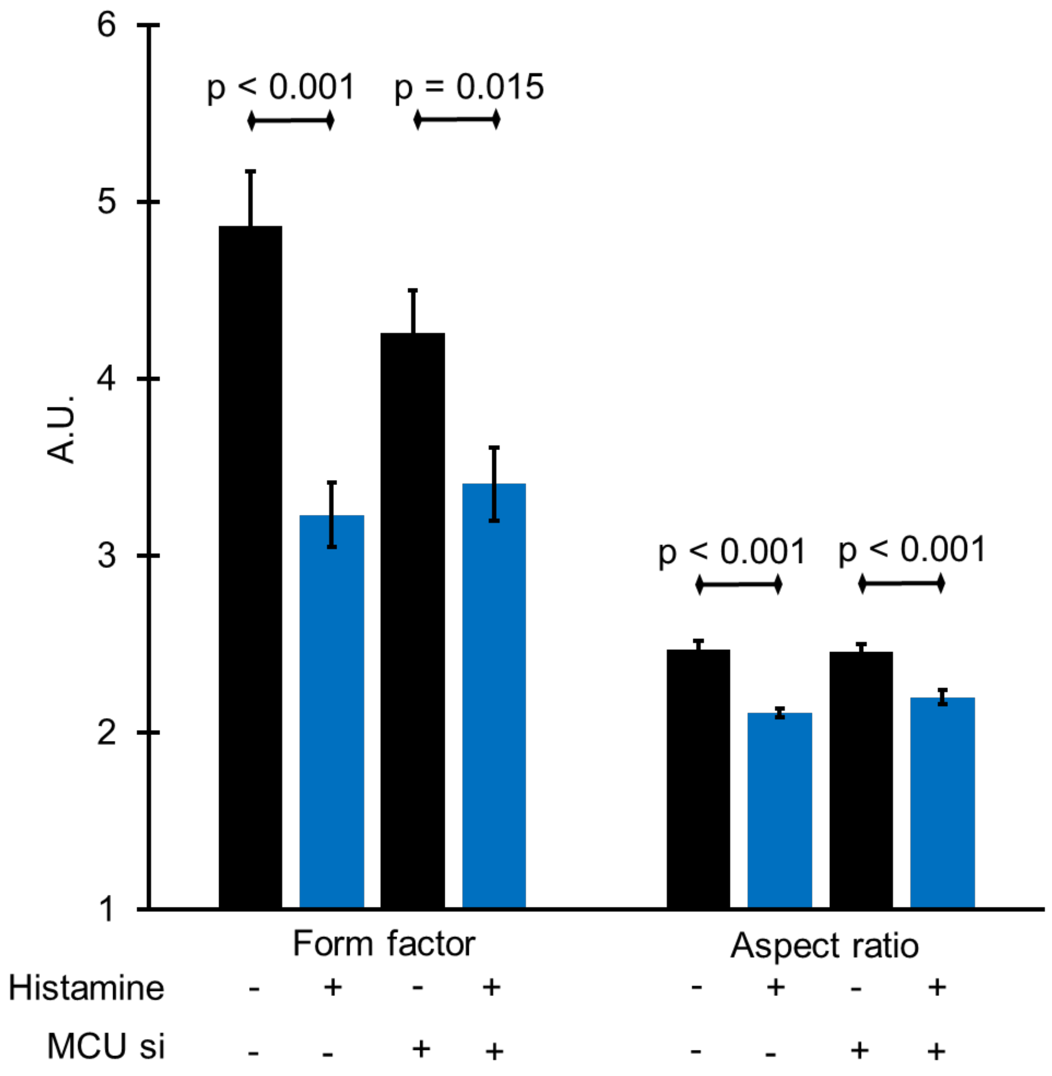

Supplementary Figure 4. HeLa cells stained with MTG and transfected with ER-RFP and control or MCU-specific siRNA were imaged with N-SIM. Histamine (100 μM) was added to induce ER Ca^{2+} release. Form factor and aspect ratio were determined using ImageJ. Images and analyses were obtained from at least 5 cells in each of 8 experiments. Bars represent mean ± SEM.

Supplementary Table 1: Sequences of the specific siRNAs used in this study

Name	Sequence
OPA1 si	5'-GUUAUCAGUCUGAGCCAGGdTdT-3'
MCU si1	5'-GCC AGA GAC AGA CAA UAC U dTdT-3'
MCU si2	5'-GGA AAG GGA GCU UAU UGA A dTdT-3'
